# Vaccine Hesitancy: Clarifying a Theoretical Framework for an Ambiguous Notion

**DOI:** 10.1371/currents.outbreaks.6844c80ff9f5b273f34c91f71b7fc289

**Published:** 2015-02-25

**Authors:** Patrick Peretti-Watel, Heidi J Larson, Jeremy K. Ward, William S Schulz, Pierre Verger

**Affiliations:** INSERM, UMR912 (SESSTIM), 13006, Marseille, France; Aix Marseille University, UMR_S912, IRD, 13006, Marseille, France; ORS PACA, Southeastern Health Regional Observatory, 13006, Marseille, France; Department of Infectious Disease Epidemiology, London School of Hygiene and Tropical Medicine, London, UK; INSERM, UMR912 (SESSTIM), 13006, Marseille, France; Université Paris Diderot, UMR8236 (LIED), 75013 Paris; Department of Infectious Disease Epidemiology, London School of Hygiene & Tropical Medicine, London, UK; INSERM, UMR912 (SESSTIM), 13006, Marseille, France; Aix Marseille University, UMR_S912, IRD, 13006, Marseille, France; ORS PACA, Southeastern Health Regional Observatory, 13006, Marseille, France; Inserm, F-Crin, Innovative Clinical Research Network on Vaccination (I-Reivac)

**Keywords:** Information Seeking Behavior, risk behavior, risk culture, vaccination behavior, vaccine hesitancy

## Abstract

Today, according to many public health experts, public confidence in vaccines is waning. The term “vaccine hesitancy” (VH) is increasingly used to describe the spread of such vaccine reluctance. But VH is an ambiguous notion and its theoretical background appears uncertain. To clarify this concept, we first review the current definitions of VH in the public health literature and examine its most prominent characteristics. VH has been defined as a set of beliefs, attitudes, or behaviours, or some combination of them, shared by a large and heterogeneous portion of the population and including people who exhibit reluctant conformism (they may either decline a vaccine, delay it or accept it despite their doubts) and vaccine-specific behaviours. Secondly, we underline some of the ambiguities of this notion and argue that it is more a catchall category than a real concept. We also call into question the usefulness of understanding VH as an intermediate position along a continuum ranging from anti-vaccine to pro-vaccine attitudes, and we discuss its qualification as a belief, attitude or behaviour. Thirdly, we propose a theoretical framework, based on previous literature and taking into account some major structural features of contemporary societies, that considers VH as a kind of decision-making process that depends on people’s level of commitment to healthism/risk culture and on their level of confidence in the health authorities and mainstream medicine.

## Related Articles

The article is part of the *PLOS Currents Outbreaks *"Vaccine Hesitancy Collection".

## Introduction

Today, according to many public health experts, public confidence in vaccines is waning^1^^,^ 2 ^,^ 3. Researchers investigating this phenomenon are now abandoning expressions such as “vaccine resistance” or “vaccine opposition”, increasingly replacing them by the new term “vaccine hesitancy” (VH) to describe the spread of vaccine reluctance. According to the Working Group on Vaccine Hesitancy appointed by the World Health Organisation’s Strategic Advisory Group of Experts (SAGE) on Immunisation, VH “refers to delay in acceptance or refusal of vaccines despite availability of vaccination services”, and this phenomenon “is complex and context specific varying across time, place and vaccines” 4. Several literature reviews have already been devoted to this issue 5 ^,^ 6 ^,^ 7 ^,^ 8. Articles using the VH terminology have mainly been published in journals specialised in vaccination or paediatric issues, but also in more generalist journals (Lancet, *British Medical Journal, *Science).*^3^^,^^9^^,^^10^*

Despite — or perhaps because of — this success, VH is an ambiguous notion and its theoretical background appears uncertain. It may have originated as a catchy expression coined by experts to capture policy-makers’ attention, as current vaccine coverage rates do not yet reflect the growing reluctance toward vaccination witnessed by empirical researchers. But hasty use of this notion may lead to misunderstandings and ineffective policies if, for example, one considers hesitancy as a necessarily transient state between full support and strong opposition.

In this paper, we first review the current definitions of VH in the public health literature and examine its most prominent characteristics; we also underline some of its ambiguities. We then call into question the usefulness of the common understanding of VH as an intermediate position along a continuum ranging from anti-vaccine to pro-vaccine attitudes, and we discuss its qualification as a belief, attitude, or behaviour. Finally, we propose a theoretical framework related to two different concepts: healthism/risk culture and (dis)trust toward health authorities and mainstream medicine.

## The ambiguities of current definitions of VH.

**VH definitions in the public health literature.**

VH has been defined as a set of beliefs 11, attitudes 8 ^,^ 12, behaviours 13, or some combination of them 11 ^,^ 12, exhibited by lay people in regard to their own or their children’s immunisations, but also sometimes by healthcare professionals. VH is an attribute ascribed to a large and heterogeneous category 5 ^,^ 6 ^,^ 7 ^,^ 11 that regroups people who share varying degrees and motives of indecision and who hold an intermediate position along a continuum ranging from full support for vaccination to strong opposition to any vaccine 5 ^,^ 6 ^,^ 11.

These people are characterised by what we may call reluctant conformism and vaccine-specific behaviours 5 ^,^ 6 ^,^ 7 ^,^ 11. They may decline a vaccine, but they may also delay it or even accept it in due time despite their doubts/reluctance. In other words, they may endorse a wide range of non-specific behaviours, all of which can result from something else than VH. This behavioural outcome can vary from one vaccine to another: they may accept one vaccine, but decline/delay another, as they base their decision on vaccine-specific features.

**A catchall category, not a concept.**

Definitions that try to describe VH as it is used by researchers tend to be very broad and to embrace heterogeneous people/situations and many different explanatory factors (including historic, political and socio-cultural contexts, as well as individual/social group influences 5 ^,^ 6 ^,^ 9). Moreover, some previous empirical studies have found quite contrasting profiles of attitudes among those who could be categorised as vaccine-hesitant 13 ^,^ 14.

Previous studies also show several contradictions in their use of VH. First, some authors argue that it is something new, distinct from old-fashioned anti-vaccination 14 ^,^ 15 ^,^ 16, while others state that it is as old as vaccination itself 6 ^,^ 12 ^,^ 17. Second, some authors consider that VH corresponds to an intermediate position, in the middle of the continuum between the pro-vaccination and anti-vaccination positions (that is, on what we shall refer to as the pro/anti continuum” 6 ^,^ 7 ^,^ 11 , while others consider that VH includes strong opposition to vaccination 18 ^,^ 19. Third, authors argue that VH is due to ignorance, misperceptions or disinformation 20 ^,^ 21, and others that it may result from too much information 12 ^,^ 17 ^,^ 22. One author even states that VH is driven basically by emotions and irrationality and vaccine refusers should be liable for the harm they cause to others 23. Fourth, VH is sometimes described with a neutral tone, or even praised 9 ^,^ 14 ^,^ 24, but it has also been described (metaphorically?) as a “condition” more or less “severe” that should be “diagnosed” among people at risk 6 ^,^ 25 ^,^ 26 and as a weed deeply rooted in the defects of human nature 20.

Finally, while some empirical studies report that VH is more frequent among people with a high socioeconomic status (SES), others report the opposite or observe no relation, especially regarding educational level 6 ^,^ 7 ^,^ 8 ^,^ 12 ^,^ 17 ^,^ 27. Such inconsistent results are disturbing for social scientists, as SES is supposed to strongly shape our beliefs, attitudes and behaviours, in meaningful ways.

These different attitudes tend to assort into specific profiles; for example, those who consider VH an old phenomenon that encompasses anti-vaccination attitudes often attribute it to ignorance, misinformation or irrationality, while those who describe it as a new attitude, distinct from strong opposition to vaccination, also argue that it is positively correlated with vaccine-related knowledge.

More generally, VH is not really an empirical concept, as the term “concept” traditionally refers to a general mental representation derived from the variety of perceived objects and defines what is common to them —the features necessary and sufficient for membership in the class/concept — through comparison, reflection and abstraction. The current definitions of VH, rather than delineating a set of core elements, cover a wide range of heterogeneous, and even sometimes contradictory, elements.

## Two issues raised by the VH definitions: what is VH, and where to position it?

**A belief/attitude, a behaviour, or a decision-making process?**

It is not fully satisfactory to define VH as a behaviour, as it is associated in the literature with various and non-specific behaviours and outcomes (due to reluctant conformism and vaccine-specific behaviours). Moreover, VH is not the only possible explanation for each of these different outcomes: acceptance might also be due to strong support, refusal to strong opposition, and delay to procrastination, oversight, or ignorance. Nevertheless, VH may be associated with other kinds of behaviours, such as information seeking, and the notion of attitude can be defined in many different ways (including a positive or negative evaluation, or a disposition to act). This ambiguity regarding the nature of VH may create problems in dealing with it: when researchers design an intervention aimed at promoting vaccination or a specific vaccine, for example, should they target an attitudinal or a behavioural outcome?

Nevertheless, beyond this ambiguity, it would be probably more useful to consider VH as a decision-making process (how/why do people come to accept/refuse/delay vaccination) which is influenced by various contextual factors (including “local vaccination cultures” 28 ^,^ 29) and leads to a variety of behavioural outcomes. From a public health perspective, behavioural outcomes are important targets in terms of prevention. But it is worth studying VH even among people who have not yet refused or delayed any vaccines, because they are more likely to do so in the future, and public health experts would like to prevent that 8.

The decision-making process we are referring to may be easy or simple (without hesitancy) or practically automatic amongst both pro-vaccine and anti-vaccine people because they have strong convictions about vaccines in general. On the contrary, this process may be difficult, more uncertain and more worthy of interest among people who have doubts — that is, who are genuinely hesitant. Thus, although VH can be considered to be an intermediate position between the pro- and anti-vaccine positions in terms of vaccine assessment (positive/negative), it is not in terms of the decision-making process. Moreover, a still different group, those with no definite opinion, little knowledge and little interest about vaccination issues and who randomly forget or delay some vaccines, share the same intermediate position on the anti/pro continuum, but they show the same behavioural pattern as people who are uncertain but very interested and committed in vaccination issues, prone to information seeking and long and balanced decision-making.

In other words, the behavioural outcomes generally associated with VH may reflect the genuine inconsistency of uncommitted people, as described above, but may also reflect a decision-making process that is not guided by a general attitude toward vaccination, but that instead takes the specificities of each vaccine/context into account, among people strongly committed to vaccination issues.

**VH and the anti/pro continuum.**

To a certain extent, keeping VH on the anti/pro continuum is convenient, as VH can be conceived as a temporary attitude of individuals who have not yet chosen between the anti- and pro- attitudes and who can more easily be convinced to endorse the latter than strong opponents 30. One might then hope to help people to move along this continuum through interventions designed to bring them what they lack: self-efficacy, knowledge or trust of the authorities.

Nevertheless, using this continuum to describe VH is not self-evident. First, from a statistical point of view, it is quite odd to choose to describe a population by representing it on an axis where most individuals are supposed to be clumped in an intermediate position. It is usually more informative to use axes that contrast individual positions.

Moreover, that vaccine-hesitant people are neither pro- nor anti-vaccination does not necessary imply that they endorse intermediate attitudes regarding vaccination in general. We can use an analogy with politics to illustrate this point. One may claim that there are hesitant voters: instead of being either leftists or rightists and always voting according to their orientation, they may make an informed choice for each election, depending on the candidates, their election platforms, and the context and territory level: they may choose to vote for a leftist candidate for the municipal election and a rightist candidate for the presidential election, and to stay home from the elections for European Parliament members. Such hesitant voters are neither leftists nor rightists, but claiming they are centrists would be highly arguable. The standard left-right clivage appears irrelevant to describing their behaviour. Similarly, the anti/pro continuum about vaccination may not be relevant for describing VH.

Finally, these people’s attitudes are claimed to be strongly vaccine-dependent, for they may accept one vaccine, be suspicious of another and strongly reject a third. Positioning them along a continuum corresponding to attitudes toward vaccination in general thus appears quite inappropriate.

## A theoretical model for VH

**The indifference/commitment axis.**

As specified above, considering VH a decision-making process helps to distinguish two very different kinds of VH: first, that of people with poor knowledge of and indifference to vaccination issues, and erratic vaccination behaviours, and, second hand, that of people who are very interested and committed to vaccination issues, prone to information seeking and long and balanced decision-making. From a psychological point of view, this axis echoes the notion of locus of control: some people believe that they can control events related to their life (internal locus of control), while others endorse a more fatalistic attitude, tending to believe that their life is driven by forces outside themselves (others, fate or luck: external locus of control) 31.

From a sociological point of view, this axis echoes two cultural features of contemporary societies, concepts known as “risk culture” and “healthism”. Risk culture is a concept coined by the British sociologist Anthony Giddens 32. According to him, people in contemporary societies are encouraged to exert autonomy over their own lives, to use available expert knowledge to stay continuously aware of risks and opportunities in their daily life, to assess risks and benefits in order to make their future secure. Authorities promote risk culture, as it is easier to govern rational and autonomous individuals, whose rationality makes them more predictable 33. This is especially true concerning health, which has become a super value: the rhetoric of self-empowerment conveyed by health promotion praises enterprising and entrepreneurial individuals who exercise control over their own behaviours and use information spread by health authorities to maximise their life expectancy. This specific cultural feature is described as healthism 34 ^,^ 35. Thus the indifference/commitment axis can also be viewed as measuring the extent to which individuals are committed or indifferent to contemporary healthism.

The link between healthism and VH is quite obvious in several studies. Gilkey et al. 26 observed that forgoing vaccines was more common among parents more attentive to their children's health and nutrition, and others underline the weight of rational assessment and the importance of perceived control among people who were reluctant to be vaccinated against the H1N1 influenza 16 ^,^ 27. Moreover, when Opel et al. 11 built a questionnaire designed to identify VH, they not only used existing items but also conducted focus groups to create new items. Because parents frequently claimed that they prefer to rely on their own research on vaccines to come to an informed decision, rather than deferring to their child’s doctor, the authors added the following item: “It is my role as a parent to question shots”. We believe this item captures an aspect of commitment to risk culture. Conversely, and rather remarkably, parents’ reluctance to get their child immunised has been used as a typical example to illustrate contemporary healthism 36.

**The trust issue.**

According to Giddens, contemporary individuals are exhorted to become the “entrepreneur” of their own life, but they must do so in a context characterised by trust issues, as many — if not all — aspects of our daily lives depend on machines or systems that are distant from us and beyond our understanding. Giddens’s analysis is based on a concept of trust borrowed from the psychologist Erik Erikson: depending on people/things that are not under our direct scrutiny or not fully understandable to us induces anxiety, and we must trust them, trust a whole expert system, through a leap of faith. As our societies are characterised by overspecialisation and the disembedding of social relationships, trust issues become essential 32. Moreover, in Giddens’s perspective, the contemporary controversies regarding vaccination or other health-related issues are not the causes of distrust, but only consequences of a wider structural phenomenon.

Trust issues are also crucial in Beck’s analyses of contemporary risk societies, especially regarding science and knowledge. According to him, our societies are characterised by reflexive scientisation: scientific scepticism has been extended to science itself and fuelled the disenchantment of science. As a result, there is a process of demonopolisation/feudalisation of scientific knowledge, with conflictual equalisation tendencies in the gradient of rationality between experts and lay people. Sciences, quasi-sciences and pseudo-sciences are competing sources that produce a flood of overspecialised, hyper-complex and contradictory findings. Consequently, distrust toward science is no longer a sign of ignorance or even obscurantism, but is endorsed by highly educated individuals 37. Beck also pointed out the increasingly important issue of conflicts of interest, i.e., situations in which scientists or experts are perceived as untrustworthy because of their financial links to industries. In this context, people who endorse risk culture and decide to take their health in hand are confronted with discordant sources of knowledge: they may distrust “official” sciences and experts, and put their faith in “alternative” sources of information or medical practice (such as homeopathy or acupuncture).

Vaccination-related issues have not escaped from these structural features of contemporary societies, especially the crisis of legitimacy faced by science, expertise and medical authorities. Vaccination may involve trust as a leap of faith, experienced through relationality and familiarity with a physician [5,38]. An annual survey conducted by the IRSN (Institute of Radiation Protection and Nuclear Safety) illustrates this crisis of legitimacy; it found that, generally speaking, the French people do not trust the way authorities manage various kinds of risks, nor do they trust the information given to them. For example, only 38% of the French had confidence that what authorities did about the H1N1 episode in 2009 was for their safety, and only 34% believed that the information released was true. 39

Trust is generally considered as a crucial component of people’s attitudes toward vaccination, including VH 5 ^,^ 7 ^,^ 8 ^,^ 16 ^,^ 40. For example, trust is a key dimension in the questionnaire developed by Opel et al to assess VH 11. More precisely, lay people may not distrust vaccines per se, but rather distrust the health authorities who are believed to be strongly influenced by vaccines producers 8 ^,^ 16. This feature of contemporary societies is closely related to healthism, and some authors have even combined these concepts: in such cases, healthism refers to individuals who seek to control their (children’s) health, who want to become its informed and rational entrepreneur, but who also express strong doubts about medical authorities and mainstream medicine and are more prone to turn to alternative experts, including on vaccination issues 36. This relation between empowerment and distrust is also explicitly promoted by some groups critical of vaccines, which claim that “trusting blindly can be the biggest risk of all” 41. Moreover, several previous studies have already found a positive correlation between VH and use of alternative medicines (such as acupuncture, homeopathy or naturopathy 16 ^,^ 17 ^,^ 40 ) , as well as distrust of colon cancer screening 14.

## VH in a two-dimension map.

Thus we propose to add to the risk culture/healthism axis a second axis, assessing lay people’s attitudes toward health authorities and mainstream medicine (see Figure 1).

**VH along two axes:**
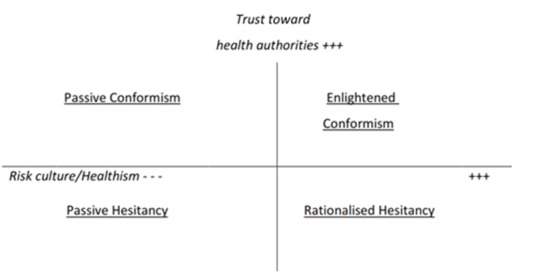
Commitment to risk culture / healthism (horizontal axis) and distrust/trust toward health authorities (vertical axis).

These two axes can be used to represent different types of VH. For example, in their study of major motives for non-acceptance of the A/H1N1 flu vaccine, Velan et al. 27 found several contrasting profiles, including people whose motives suggested passiveness, inaction and dependence (“I did not think about it”, “I somehow did not manage to do it”...), corresponding here to the lower-left quadrant (erratic VH), and people who made their decision after a process of reflection (“after some deliberation I have decided not to do it”, “I realised that the disease is not serious, that it is not spreading”), corresponding here to the lower-right quadrant. Specific views regarding VH could also be positioned here: for example, in Marshall’s view 20, VH is due to a lack of faith in authorities, combined with misperceptions, emotion-driven reactions, and gullibility toward rumours spread by anti-champions and celebrities, corresponding to the lower-left quadrant.

This map also underlines that although health promotion policies aiming at encouraging people to take their health in their own hands may move people further along the horizontal axis, health promotion may nonetheless fuel VH, at least among some populations, unless they are positioned high on the trust axis.

Nevertheless, talking about vaccine hesitant people may be a misuse of language, as VH also strongly depends on vaccine-specific factors; thus specific vaccines, rather than individual profiles, could be also positioned along our two axes. In other words, instead of considering whether or not people are vaccine-hesitant, we should consider whether a specific context/vaccine fuels their propensity to VH. For example, people are probably more likely to endorse an active attitude, seeking information and assessing risk and benefits, when they are confronted with new vaccine, such as the H1N1 or the HPV vaccines, while they may be more prone to follow their physician’s advice routinely and passively for some childhood vaccines or that against seasonal influenza. Moreover, the level of confidence in health authorities about any specific vaccine obviously depends on the extent of media coverage of existing controversies regarding its potential side effects. In the UK, for example, the MMR vaccine would have been positioned in the upper-left quadrant before the MMR/autism controversy began, along with the vaccine against seasonal flu. On the contrary, the HPV vaccine could be considered as relatively new and less controversial (upper-right quadrant), while the H1N1 vaccine was both new and controversial (lower-right quadrant) 15.

## Conclusion

In this paper, we have discussed some of the ambiguities and contradictions of the notion of vaccine hesitancy. We have argued that it is currently more a catchall category than a real concept. This lack of consistence is likely to hamper both research and interventions. Therefore, as a supplement to the previous work carried out by the SAGE working group, we propose grounding the notion of vaccine hesitancy in an explicit theoretical framework that takes some major structural features of contemporary societies into account. We consider VH to be a kind of decision-making process that depends on people’s level of commitment to healthism/risk culture and on their level of confidence toward health authorities and mainstream medicine.

This framework abandons the anti/pro vaccination continuum. We do not claim here that the vaccine-hesitant do not have opinions about vaccination in general, nor that such opinions do not influence their behaviours toward a specific vaccine. Nevertheless, we believe that these general opinions are not a key determinant of their vaccination behaviours, especially in comparison with the two other dimensions discussed here.

We also believe our approach opens new avenues for research on VH. For example, it would be useful to consider various kinds of VH that may be correlated to individuals’ socioeconomic status (SES), as the social differentiation of health behaviors may reflect the social differentiation of health-related cognition, including knowledge, attitudes and beliefs **** 42. Erratic VH is more likely to be displayed by people with low SES, while rationalised VH may be more frequent among the educated middle classes 43.

Finally, as even highly educated people may show VH, we should not focus only on lay people. We should also study extensively VH among health professionals, and especially among general practitioners, who still play a key role in promoting vaccination, and who also display some kinds of VH 5 ^,^ 8 ^,^ 17. We should also study their interactions with their patients. In some cases, health professionals may fuel patients’ VH (directly, when homeopaths and naturopaths express convincingly their doubt regarding vaccination to their patients 40, or indirectly, when pro-vaccine physicians refuse to accept into their practices families who are reluctant to vaccinate their children 25), but sometimes vaccine-hesitant patients may raise doubts in their physician’s mind 38.

More generally, we should study the interactions between the various participants involved in immunisation policies, from public health experts to lay people: is there a ‘hesitancy cascade’, with waves of influences from political hesitancy to provider hesitancy to public hesitancy, and is there a feedback effect in some cases (patients’ VH reinforcing physicians’ VH, which in turn fuels VH among other patients)?

## Competing Interests

The authors have declared that no competing interests exist.
